# Network changes associated with right anterior temporal lobe atrophy: insight into unique symptoms

**DOI:** 10.1093/braincomms/fcaf251

**Published:** 2025-06-24

**Authors:** Hulya Ulugut, Maria Luisa Mandelli, Anna Gilioli, Zoe Ezzes, Janhavi Pillai, David Baquirin, Amie Wallman-Jones, Amanda Gerenza, Eleanor R Palser, Aaron Scheffler, Giovanni Battistella, Yann Cobigo, Howard J Rosen, Zachary A Miller, Kyan Younes, Bruce L Miller, Joel Kramer, William W Seeley, Virginia E Sturm, Katherine P Rankin, Maria Luisa Gorno-Tempini

**Affiliations:** Memory and Aging Center, Department of Neurology, University of California, San Francisco, CA 94158, USA; Memory and Aging Center, Department of Neurology, University of California, San Francisco, CA 94158, USA; Memory and Aging Center, Department of Neurology, University of California, San Francisco, CA 94158, USA; Neuroimaging Research Unit, Division of Neuroscience, IRCCS San Raffaele Scientific Institute, Milan 20132, Italy; Memory and Aging Center, Department of Neurology, University of California, San Francisco, CA 94158, USA; Memory and Aging Center, Department of Neurology, University of California, San Francisco, CA 94158, USA; Memory and Aging Center, Department of Neurology, University of California, San Francisco, CA 94158, USA; Memory and Aging Center, Department of Neurology, University of California, San Francisco, CA 94158, USA; Memory and Aging Center, Department of Neurology, University of California, San Francisco, CA 94158, USA; Memory and Aging Center, Department of Neurology, University of California, San Francisco, CA 94158, USA; Department of Psychology, Palo Alto University, Palo Alto, CA 94304, USA; Department of Epidemiology and Biostatistics, University of California, San Francisco, CA 94158, USA; Department of Otolaryngology-Head and Neck Surgery, Massachusetts Eye and Ear and Harvard Medical School, Boston, MA 02114, USA; Memory and Aging Center, Department of Neurology, University of California, San Francisco, CA 94158, USA; Memory and Aging Center, Department of Neurology, University of California, San Francisco, CA 94158, USA; Memory and Aging Center, Department of Neurology, University of California, San Francisco, CA 94158, USA; Department of Neurology and Neurological Sciences, Stanford University, Stanford, CA 94304, USA; Memory and Aging Center, Department of Neurology, University of California, San Francisco, CA 94158, USA; Memory and Aging Center, Department of Neurology, University of California, San Francisco, CA 94158, USA; Memory and Aging Center, Department of Neurology, University of California, San Francisco, CA 94158, USA; Memory and Aging Center, Department of Neurology, University of California, San Francisco, CA 94158, USA; Memory and Aging Center, Department of Neurology, University of California, San Francisco, CA 94158, USA; Memory and Aging Center, Department of Neurology, University of California, San Francisco, CA 94158, USA

**Keywords:** anterior temporal lobe, social semantics, mental rigidity, functional connectivity, frontotemporal dementia

## Abstract

Semantic behavioural variant (also referred to as right temporal) frontotemporal dementia is a newly described syndrome associated with predominant right anterior temporal lobe atrophy and a distinctive combination of behavioural and semantic changes. It is considered the right-sided counterpart of semantic variant primary progressive aphasia, with which it has overlapping neuropathological and cognitive mechanisms. Although more is known about how brain network alterations relate to both losses (e.g. word comprehension deficits) and increases (e.g. hyper-fluency) in cognitive and behavioural processes in the left-sided semantic progressive aphasia, less is known about these phenomena in the right-sided semantic behavioural variant. In this study, we investigated functional connectivity within the right counterparts of established ventral and dorsal cortical speech and language networks and their relationship to specific clinical manifestations in individuals with the semantic behavioural variant. We hypothesized that socioemotional-semantic deficits would be associated with reduced connectivity in the right ventral semantic network, while heightened behavioural manifestations, such as hyper-focus on specific interests (also referred to as rigidity), would be associated with increased connectivity in the right dorsal network. Using seed-based intrinsic connectivity analyses of functional MRI data and cognitive scores from 22 individuals with semantic behavioural variant frontotemporal dementia and 48 cognitively normal individuals, we measured intrinsic connectivity strength in networks anchored in the right anterior middle temporal gyrus (ventral network) and in the right opercular inferior frontal gyrus (dorsal network). Functional connectivity values were then correlated with cognitive and behavioural measurements, controlling for global atrophy. Compared to the control group, individuals with semantic behavioural variant exhibited reduced connectivity in the right ventral network (*t* = 2.7, *P* = 0.003), which was associated with socioemotional-semantic deficits (*r* = 0.47, *P* = 0.03), as measured by standardized tests. Conversely, increased functional connectivity was observed in the spared right dorsal network (*t* = 4.26, *P* < 0.001), which correlated with higher scores for hyper-focus on fixed interests, as measured by retrospective ratings of medical notes (*r* = 0.63, *P* = 0.002). Together with previous evidence, these findings suggest that in individuals with predominant anterior temporal lobe atrophy, greater expression of behaviours such as hyper-focus is associated with altered functional dynamics within networks that remain relatively spared by the disease process. This highlights the complex interplay between damaged and spared networks in shaping the clinical manifestations of semantic behavioural variant frontotemporal dementia.

## Teaser

Ulugut and Mandelli et al. used functional brain imaging and clinical data to investigate rare right-lateralized frontotemporal dementia. They found network disruptions linked to socio-emotional deficits and compulsive narrow interests. Results suggest symptoms emerge from dynamic interactions between damaged and intact neural circuits, not from injury to single, isolated brain regions.

## Graphical Abstract

**Graphical Abstract:**
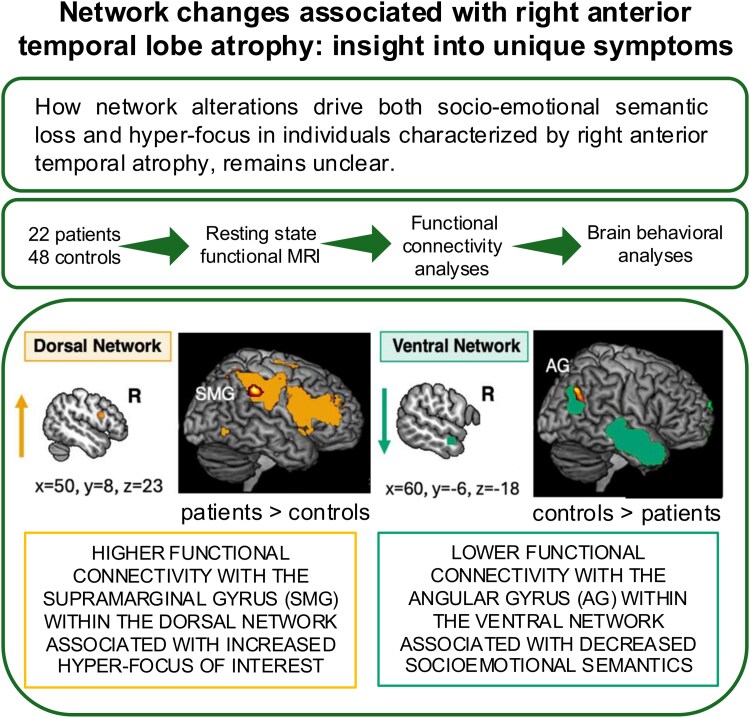

## Introduction

Frontotemporal dementia (FTD) is one of the leading causes of dementia before 65 years of age and most often manifests as behavioural problems [in behavioural variant FTD (bvFTD)]^1^ or speech and language impairment [in primary progressive aphasia (PPA)].^2^ These symptoms arise from neurodegeneration affecting either the frontal or temporal lobes, or both.^1-3^ Behavioural problems in FTD are primarily characterized by interpersonal socioemotional deficits, which vary depending on the brain regions affected.^1,4^ The term semantic behavioural variant frontotemporal dementia (sbvFTD)—also referred to as right temporal FTD^5-8^ or right semantic dementia (SD)^9-11^—describes a clinical syndrome associated with predominant atrophy in the right anterior temporal lobe (rATL).^12^ Current cognitive neuroscience and clinical descriptions of sbvFTD indicate that the rATL serves as a semantic hub for socially and emotionally relevant information, such as knowledge of famous people, the meaning of facial expressions and paralinguistic cues, and other social concepts and norms.^5,6,12-16^ Deficits in these semantic functions most often manifest clinically as loss of empathy,^4,10,12,16-20^ with patients exhibiting difficulty understanding social situations and emotions, leading them to be unattuned or unresponsive to others’ needs and emotional states.

A substantial body of work shows that both hemispheres contribute to language and socioemotional skills, yet each is relatively biased towards different facets of semantic processing. Studies on neurodegenerative disease and functional MRI in healthy controls (HCs) converge on the ventral temporo-parietal network as the key substrate for lexical and object-related semantic processing, especially—though not exclusively—in the left hemisphere. For instance, in the left-hemisphere counterpart of sbvFTD, termed semantic variant PPA (svPPA), structural and functional connectivity (FC) changes are associated with deficits in word and object identification and are related to multimodal loss of semantic knowledge for objects.^2,21-27^ Similarly, increasing evidence links right ventral network damage to semantic processing deficits for famous people and socioemotional information.^12,13,19,28-31^ Disease-related degeneration of the ATL, a key hub in the temporo-parietal network, has been associated not only with cognitive and behavioural deficits but also with ‘heightened’ behaviours, such as increased speech rate (referred to as hyper-fluency or press of speech) in individuals with svPPA.^32^ However, individuals with sbvFTD with more right-lateralized degeneration show a hyper-focus on fixed interests (HFIs).^19^ This behavioural hyper-focus manifests across a spectrum of activities, from artistic pursuits to repetitive behaviours like clock-watching.^5,6,9^ These symptoms have been regularly described over the years, but are heterogeneously labelled as rigidity, obsessions, compulsions, repetitive rituals, or preoccupations,^5,7,9,12,33^ or more specifically as hyper-religiosity^34,35^ or hyper-graphia.^35,36^ Recent retrospective work from an international group of experts reported that 78% of sbvFTD patients exhibit such behaviours at early stages of the disease.^6^ Despite their high prevalence and relevance to everyday functioning, theoretical models focusing on the rATL do not fully explain the emergence of these behaviours in relation to structural atrophy. Some researchers suggest these behaviours stem from disturbances in reward processing, with a shift in hedonic valuation due to altered direct connectivity between the ATL and medial orbitofrontal regions.^14,37^ Others attribute these behaviours to semantic loss, suggesting that deficits in comprehending multimodal (e.g. visual, auditory and chemosensory) socioemotional stimuli limit individuals’ options, leading to stronger personal preferences for stimuli that remain comprehensible to them.^38-40^ While these hypotheses may account for the narrowness of interests, they do not explain why these activities become central to patients’ daily lives or why these actions are performed repeatedly with heightened motivation and extended attention spans (e.g. spending over eight hours each day writing^36^).

Although traditional lesion-based correlation studies have been instrumental in identifying symptoms associated with focal brain damage, such as linking ATL damage to semantic deficits in disorders affecting this region,^13,41,42^ they have been insufficient for elucidating the neural mechanisms underlying clinical manifestations like HFIs. Studies using task-free functional MRI (tf-fMRI)^43^ have allowed the investigation of FC in neurodegenerative diseases^44-46^ with selective vulnerability to specific brain networks.^47,48^ These studies provide evidence that degeneration in a critical network node can induce large-scale network-level changes,^49,50^ potentially offering new insights into unique symptomatology. For instance, functional neuroimaging studies in svPPA revealed functional alterations and clinical correlations within left-hemisphere networks anchored in regions crucially involved in the disease.^24,32^ Decreased FC in the ventral semantic network [seeding from the anterior portion of the middle temporal gyrus (aMTG) to the angular gyrus (AG)] correlated with verbal semantic deficits, the hallmark of svPPA. Conversely, increased FC in the left dorsal frontal network [seeding from the opercular part of the inferior frontal gyrus (opIFG) to the supramarginal gyrus (SMG)] was associated with an increased articulation rate.^32^ These findings provide a compelling model for investigating altered functional responses to focal structural neurodegeneration in sbvFTD.

Substantial evidence links the right ventral network damage to socioemotional semantics, but the right dorsal frontal network, anchored in opIFG, has received less attention despite its putative role in sustaining task-set, allocating attention, and modulating reward responsiveness. Resting-state FC studies in dementia suggest that decreased FC in this network is associated with attention deficits and apathy.^51,52^ However, beyond the dementia literature, a large body of research in healthy individuals and those with neurodevelopmental disorders has indicated that increased FC in this network correlates with heightened task-related attentional load, action execution, rule acquisition, and enhanced responsiveness to positive outcomes,^53-58^ resulting in clinical outcomes described as ‘attentional strengths’, ‘hyper-focus’ or ‘difficulties shifting attention’.^53-60^ These findings have not been tested in patients with dementia and such network alteration may explain symptoms such as HFIs commonly seen in sbvFTD.

In this study, we utilized seed-based tf-fMRI alongside clinical, cognitive and behavioural data from a cohort of individuals with early-stage sbvFTD and HCs. We hypothesized that sbvFTD would cause FC alterations akin to those observed in svPPA within their ventral and dorsal right counterparts. Specifically, we hypothesized that the anatomical (right-side) counterpart of the ventral semantic network would show decreased FC, correlating with socioemotional-semantic skills deficits, and that the counterpart of the frontal-dorsal network would show increased FC, correlating with higher HFI scores. This design aimed to provide new insights into the unique symptom combinations typical of sbvFTD.

## Materials and methods

### Participants selection

Patients with a clinical diagnosis of bvFTD,^1^ svPPA,^2^ SD^3^ and/or sbvFTD^12^ were identified from a database of individuals who participated in a prospective, multidisciplinary study on FTD at the Memory and Aging Center, University of California, San Francisco (MAC-UCSF). Patients with predominant rATL atrophy were identified using the atrophy lateralization index, as detailed in our previous publication.^12^ Patients were excluded if (i) they did not have a resting-state fMRI sequence (*n* = 30); (ii) their Mini-Mental State Examination (MMSE)^61^ scores were <20 or clinical dementia rating (CDR)^62^ scores were ≥2 (*n* = 2); (iii) structural MRI motion artefacts (*n* = 5), white matter hyperintensities (Fazekas >2)^63^ (*n* = 4), or fMRI artefacts (*n* = 2) were present.

Neurologically healthy, community-dwelling older adults (*n* = 48) were recruited as a control group from a cohort enrolled in the BRain Aging Network for Cognitive Health study at the MAC-UCSF. Participants were verified as neurologically normal through a multidisciplinary assessment, which included neurological examination, neuropsychological and cognitive testing, and neuroimaging evaluation.

All participants, or their primary caregivers provided written informed consent for their participation in the study, and the experimental procedures were approved by the UCSF Human Research Protection Program.

### Functional, cognitive and behavioural assessments

Each participant completed a comprehensive multidisciplinary evaluation that included neurological, functional, cognitive and behavioural measures, as well as neuroimaging assessment.^64^ All data were collected within an average 90-day window before or after fMRI scanning. The characteristics of the sample are summarized in Table 1. Descriptions of the assessment tools listed in Supplementary Table 1 are provided in our previous publications.^12,64-66^

**Table 1:** Demographics and functional scores

	sbvFTD	HC
Demographics and functional scales	*N* = 22	*N* = 48
Age at evaluation (years), mean (SD)	65.5 (6.7)	65.7 (7.2)
Sex (Female %)	50%	50%
Education (years), mean (SD)	16.6 (3.0)	17.5 (2.0)
Handedness (right/left/ambDx)	19/1/2	46/2/0
Scanner Type (Trio/Prisma)	12/10	24/24
CDR score, mean (SD), max = 3	0.8 (0.4)	0 (0)
CDR Box score, mean (SD), max = 18	4.7 (3.1)	0 (0)
FTLD Box	6.7 (3.7)	0 (0)
Global cognition		
MMSE, mean (SD), max = 30	26.1 (2.9)*	29.3 (0.8)

sbvFTD, semantic behavioural variant frontotemporal dementia; HCs, healthy controls; CDR, Clinical Dementia Rating; MMSE, Mini-Mental State Examination; SD, standard deviation.

^*^sbvFTD different from health controls at *P* < 0.05.

### Cognitive and behavioural scores for functional imaging correlations

For the brain-behavioural correlation analyses, we selected cognitive and behavioural measures that, accordingly to prior literature, map onto the ventral or dorsal networks. Specifically, we hypothesized that decreased connectivity in the right ventral network would correlate with lower scores on tests assessing semantic processing of famous people and socioemotional concepts^13,16,28^ and that decreased connectivity in the left ventral network would correlate with poorer verbal semantic skills. To quantify these constructs, we created two composite scores associated with socioemotional semantics and verbal semantic skills, respectively, for the right and left ventral networks: (i) for socioemotional semantics, the UCSF Famous Face Battery (FFB),^29^ which includes confrontation naming, semantic association, and familiarity judgment tasks, and the Comprehensive Affect Testing System Affect Matching (CATS-AM) test^67^; (ii) for verbal semantics, the Boston Naming (BNT) and the Peabody Picture Vocabulary Tests (PPVT). Composite scores were calculated as the mean of proportion of maximum scores for the constituent tests as the following:


$$\mathrm{Composite}=\frac{\{(\mathrm{scor}e_{1}/\mathrm{ma}x_{1})+(\mathrm{scor}e_{2}/\mathrm{ma}x_{2})+\ldots +(\mathrm{scor}e_{n}/\mathrm{ma}x_{n})\}}{n}.$$

In previous studies on svPPA, increased connectivity in the left dorsal speech production network was linked to higher speech rate, suggesting that increased connectivity in spared networks may drive ‘positive’ symptoms such as press of speech or hyper-fluency.^32^ Drawing from the sbvFTD/right temporal literature, we proposed that HFIs, often termed rigidity or compulsive behaviours, might similarly be linked to increased dorsal connectivity.^19,52,57,58^ Given the lack of a standardized assessment tool to quantify the presence or the severity of these symptoms, we devised a scoring protocol based on case notes.

Case notes were independently reviewed by four clinical researchers (H.U., Z.M., A.G., A.W.J.) to determine the presence or absence of HFI symptoms. The scoring framework was based on the well–established Neuropsychiatric Inventory (NPI)^68^ algorithm, which multiplies symptom frequency by severity. Adapting this structure to our context, we calculated an HFI score by multiplying the variety of activities by their frequency, paralleling the NPI’s frequency × severity logic. After the initial ratings, reviewers cross–checked each other’s notes and resolved any discrepancies through team consensus. Variety was coded as 1 when activities were confined to a single domain (e.g. only cleaning) and 2 when they spanned multiple domains (e.g. cleaning, writing, religious practice). Frequency was coded as 1 for occasional occurrences (e.g. once a week, not part of the daily routine) and 2 for activities integrated into the daily routine. Absence of HFI symptoms yielded a score of 0. Examples of chart-review excerpts and their corresponding ratings are provided in Supplementary Table 2.

#### Rationale and scoring safeguards

We substituted *Variety* for the NPI’s *Severity* dimension because case notes rarely documented symptom intensity, whereas clinical experience indicates that interests spanning multiple domains are typically more disruptive. To avoid artificially inflating scores, Variety and Frequency were always calculated within the same HFI domain—the frequency of one interest was never multiplied by the variety of another. Episodes of extreme duration (e.g. ≥ 9 h day⁻¹) were coded under the ‘daily routine’ category (Frequency = 2), capturing the repetitive nature of the behaviour without overstating sparse quantitative detail. The resulting composite score (0–4) was treated as an ordinal variable in all statistical analyses, with higher values reflecting progressively greater symptom burden.

### Brain MRI dataset

#### MRI acquisition

MRI images were acquired at the UCSF Neuroscience Imaging Center using two 3T Siemens scanners (Prisma or Trio). Magnetization-prepared rapid acquisition with gradient echo acquisition (MPRAGE) was used to acquire T1-weighted images. Parameters were similar for both scanners: 160 sagittal slices, isotropic voxel size of 1mm³, repetition time (TR) of 2300 ms, echo time (TE) of 2.98 ms, inversion time of 900 ms, flip angle of 9°, a field of view (FoV) of 256 × 256 mm^2^, and an integrated parallel acquisition technique (iPAT) acceleration factor of 2. For fMRI, participants were instructed to lie still with closed eyes, ensuring they stayed awake. T2*-weighted volumes were acquired using an echo planar imaging (EPI) protocol. The Trio scanner captured 240 volumes of 36 AC/PC-aligned axial slices in an interleaved order, with TR/TE of 2000/27 ms, flip angle of 80°, slice thickness of 3 mm with a 0.6 mm gap, pixel size of 2.5 × 2.5 mm^2^, field of view of 230 × 230 mm, and matrix of 92 × 92. The Prisma scanner captured 560 volumes of 66 slices with similar alignment, TR/TE of 850/32.8 ms, flip angle of 45°, slice thickness of 2.2 mm, pixel size of 2.2 × 2.2 mm^2^, field of view of 211 × 211 mm^2^, matrix of 96 × 96, and a multi-band acceleration factor of 6. Additionally, two volumes were acquired for distortion correction, using b = 0 and opposite phase encoding directions (anterior/posterior and posterior/anterior).

#### Brain structural MRI preprocessing

**Figure 1:**
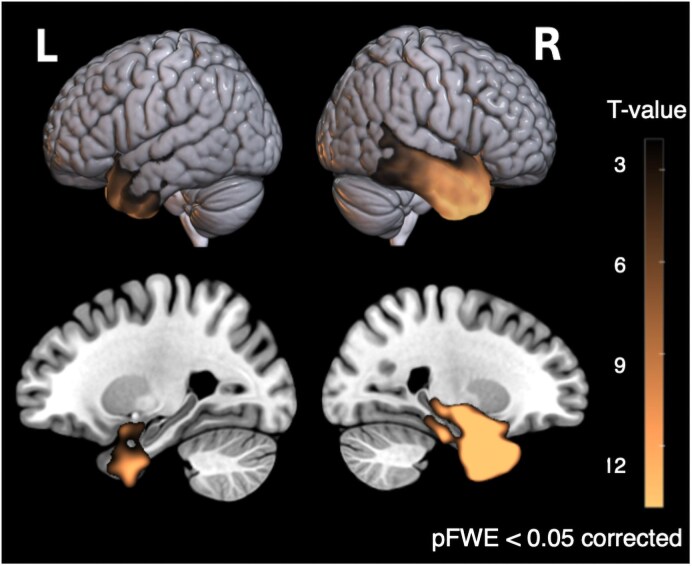
**Grey matter atrophy in sbvFTD.** Voxel-based morphometry of structural MRI shows maximal volume loss in the right anterior temporal lobe compared with HCs. Group differences were tested with a GLM in SPM12, controlling for age, sex, handedness, and TIV; voxels meeting *P* < 0.05, FWE correction are displayed on the MNI template (R = right, L = left). Sample sizes: HC = 48, sbvFTD = 22. Rendering produced in MRIcroGL. sbvFTD, semantic behavioural variant frontotemporal dementia; HCs, healthy controls; GLM, general linear model; SPM, Statistical Parametric Mapping; TIV, total intracranial volume; FWE, family-wise error; MNI, Montreal Neurological Institute.

Structural MRI data were processed using the Computational Anatomy Toolbox (CAT12) within the Statistical Parametric Mapping software framework (SPM12), running on Matlab 2021b. The data were enhanced using a spatial adaptive non-local means denoising algorithm,^69^ followed by bias field correction, affine transformation for alignment, and processing using SPM's ‘unified segmentation’ protocol.^70^ The images were segmented into grey matter, white matter, and cerebrospinal fluid, and then (i) spatially normalized to the Montreal Neurological Institute (MNI) reference space via an advanced geodesic shooting technique,^71^ (ii) adjusted by the Jacobian determinants of the deformation field during spatial normalization to preserve tissue volume integrity, and (iii) output with a uniform isotropic voxel resolution of 1.5 × 1.5 × 1.5 mm^3^. Spatially normalized, segmented, and modulated grey matter images were finally smoothed using an 8 mm FWHM isotropic Gaussian kernel. Voxel-based inferential statistic was performed by fitting a general linear model in SPM12 on the smoothed and modulated GM tissue probability maps entering age, sex, handedness and total intracranial volume (TIV) as covariates of no interest. The statistical map showing grey matter volume differences between HC and sbvFTD was thresholded at *P* < 0.05 family-wise error (FWE) corrected. The creation of the atrophy maps is further detailed in our previous publications.^72,73^ The results of the atrophy maps are illustrated in Fig. 1.

### Functional MRI data

#### Preprocessing of task-free fMRI

Functional MRI data were processed using a custom pipeline developed in Python, incorporating components from the Brain Software Library for Functional MRI (FSL), Statistical Parametric Mapping (SPM), Advanced Normalization Tools^74^ and the Analysis of Functional NeuroImaging (AFNI).^75,76^ To facilitate T1 equilibrium, the initial five volumes were excluded. The remaining volumes were slice-time corrected, realigned to the mean functional image, and assessed for rotational and translational head motion. EPI-related distortion artefacts were mitigated using FSL's TOPUP utility, leveraging two acquired images with opposing phase-encode blip directions. The functional volumes were then registered to an EPI template in the MNI space using a combination of linear and non-linear warping. Spatial smoothing was applied using a 5-mm FWHM Gaussian kernel. Finally, the functional data were bandpass filtered within the frequency range of 0.008 Hz < *f* < 0.15 Hz, and non-signal fluctuations, including motion artefacts, their derivatives, and quadratic terms, along with white matter and cerebrospinal fluid signals, were regressed from the functional data as outlined in Satterthwaite and colleagues’ research.^77^

Subjects were included only if inter-frame head translations were <2 mm, head rotations were <2°, and motion spikes (defined as inter-frame head displacements greater than 1 mm) occurred in <5% of the total frames. Based on these criteria, five participants were excluded due to excessive motion or the presence of artefacts, and two participants were excluded due to white matter hyperintensity that could alter the interpretation of FC. The final cohort consisted of 22 individuals with rATL and 48 HCs.

#### Definition of seed-based FC analysis

The seed ROIs were defined as spheres of 5-mm radius centered at (i) the right anterior middle temporal gyrus (aMTG; MNI coordinates: *x* = 60, *y* = −6, *z* = −18) and (ii) the pars opercularis of the inferior frontal gyrus (opIFG; MNI coordinates: *x* = 50, *y* = 8, *z* = 23) (Fig. 1). To validate hemispheric specificity, we repeated the analysis with homologous left-hemisphere seeds at *x* = −60, *y* = −6, *z* = −18 (aMTG) and *x* = −50, *y* = 8, *z* = 23 (opIFG). For each seed, single-subject correlation maps were generated by calculating the *r*–Pearson correlation coefficient between the mean blood-oxygen-level-dependent signal time course of each seed ROI and the time course from all other voxels of the brain. The *r*-correlation maps were then Fisher *z*-transformed for parametric comparisons.

The network anchored to the opIFG seed (right dorsal) included areas in the bilateral opercular and triangular part of the IFG, right middle frontal gyrus, right SMG, bilateral supplementary motor area and right inferior temporal gyrus (ITG). The network anchored to the aMTG seed (right ventral) included areas in the bilateral temporal pole, ITG, precuneus, hippocampus, and parahippocampal gyrus. Additional significant regions were located in the right AG, anterior insula, orbital IFG, bilateral anterior and posterior cingulate cortices, as well as in the bilateral orbital medial frontal gyrus. Networks derived from the left–hemisphere seeds showed the expected mirror–symmetric topography, as previously described.^23^

#### Harmonization of functional MRI data

To mitigate biases introduced by the use of different scanners and sequence parameters, we applied the ComBat method^78,79^ to the functional correlation maps after they were transformed into *z*-scores. This approach utilizes a multivariate linear mixed-effects regression that incorporates terms for both biological variables (age and sex) and scanner types to adjust for imaging feature measurements. Additionally, ComBat employs empirical Bayes techniques to refine the estimation of model parameters, which is particularly beneficial in studies with limited sample sizes. ComBat harmonization analyses were conducted using a publicly available R package, accessible at https://github.com/Jfortin1/ComBatHarmonization.

### Statistical analyses

#### Demographic and clinical data

Descriptive statistics were calculated for all available demographic and clinical data. Between-group differences were assessed using t-tests for continuous variables and chi-square tests for categorical variables. All analyses were conducted using Python (version 3.10.9), with the significance level set at *P* = 0.05. Pearson correlation analyses were performed to assess the linear association between the variables of interest. To mitigate the potential impact of disease severity or global cognitive impairment, participants with a CDR ≥ 2 and/or an MMSE < 20 were excluded (see the participant selection section), thus such covariates were not included in the analyses.

#### Analysis of functional MRI data

**Group comparisons**: Single-subject FC maps for each seed-based networks were entered into a voxel-wise two-sample *t*-test in SPM12 to identify FC alterations between the healthy cohort and the cohort of individuals with rATL. The cortical volume of the corresponding seed ROIs was included as covariates of no interest (age and sex are already modelled by ComBat). We included the total cortical volume as a measure of global atrophy. An implicit (binarized) mask of the networks of interest, defined at group level was used as previously defined in Battistella *et al.* 2020^23^ (after FWE correction for multiple comparisons at peak level and *k* > 180 for cluster extent in a healthy group of controls).

**Brain-behaviour Correlations:** We conducted one-sided Pearson correlation analyses to examine specific directional hypotheses. We hypothesized that increased FC in the spared right dorsal network would be positively correlated with higher HFI scores, and that decreased FC in the damaged right ventral network would be positively correlated with lower scores on non-verbal socioemotional measures (FFB and CATS-AM). For completeness, we also correlated FC in the left ventral network with verbal–semantic performance (BNT + PPVT). There were no FC alterations in the left dorsal network, therefore no brain-behaviour correlation analyses were conducted. The one-sided test aligns with our directional hypotheses, consistent with prior evidence and theoretical expectations linking FC to behavioural outcomes. While there were no missing data for HFI, three participants had missing data for each of the FFB tasks. To ensure robustness, we performed the analyses both excluding these three participants and using an imputation method to address the missing values. The imputation analysis was conducted for sensitivity purposes using multiple imputation (*m* = 20) with parameter estimates pooled according to Rubin’s rules. Detailed procedures for the imputation analysis are provided in the Supplementary material.

## Results

### Description of the cohort of patients

Table 1 summarizes the demographics and functional scales scores of the two cohorts. The healthy control group was demographically matched to the sbvFTD group; therefore, the cohorts did not differ significantly in sex, age, or education. Eighteen percent of sbvFTD participants were left-handed or ambidextrous. Clinical symptoms were consistent with the distribution of cortical atrophy, and all patients exhibited the same hemispheric lateralization pattern as the right-handed participants. Mean MMSE score was lower in sbvFTD (27 ± 2.7) than in controls (29.8 ± 0.3), in line with published norms.^61^ Mean CDR was 0.8 ± 0.4, indicating early-stage disease. Additional cognitive and behavioural data are provided in Supplementary Table 1. Attention, calculation, and visuospatial skills were preserved, and no rule violations occurred on verbal (phonemic/categorical) or design fluency tasks. Executive functions were generally within normal limits, except for mild to moderate impairments on design fluency (*P* = 0.02) and Trails tests (time, *P* = 0.03). As expected, sbvFTD patients exhibited severe impairments (*P* < 0.001) in visual semantic knowledge (measured by the Pyramids and Palm Trees pictures test) as well as verbal semantic knowledge (measured by the BNT and PPVT). They also showed deficits in person-specific knowledge (measured by the FFB) (*P* < 0.001), although face perception skills remained intact. Affective knowledge was impaired, as indicated by performance on the CATS-AM, Dynamic Affect Recognition Test, and the Awareness of Social Inference Test (TASIT) Emotion Evaluation Test (*P* < 0.001). Additional deficits were observed in lexical and verbal fluency, episodic memory, and recognizing paralinguistic cues (on the TASIT-sarcastic sections, while comprehension of sincere conversations was intact) (*P* < 0.001). Emotional perspective-taking was impaired (on the emotional Theory of Mind [ToM] test) (*P* < 0.001), although cognitive ToM was within normal limits. Informant-based surveys indicated diminished emotional sensitivity and reduced interpersonal reactivity, particularly in empathic concern and perspective-taking (*P* < 0.001). Chart review ratings revealed markedly increased motivation and attention towards specific routine activities, many of which were part of their daily routine, often pursued for hours daily. Examples of this HFI are listed in Supplementary Table 2.

### Atrophy profile in sbvFTD

The atrophy distribution exhibits the expected bilateral pattern within the temporal lobes, with the most significant peak observed in the rATL (MNI, *x* = 22, *y* = 3, *z* = −44; *T*-value = 22.22, cluster size = 108,072, *P* < 0.05, FWE-corrected). Atrophy is most prominent in the ITG, middle temporal gyrus (MTG), entorhinal cortex, and fusiform gyrus, extending to the orbital gyrus and amygdala (Fig. see Supplementary Table 3 for local peak coordinates). Brain rendering were created with MRICroGL (https://www.nitrc.org/projects/mricrogl).

### Functional connectivity

#### Altered FC in sbvFTD

Within the network seeded in the right opIFG, sbvFTD patients showed increased FC compared to HCs in a cluster localized in the right SMG (MNI coordinates of the cluster peak: 60, −32, 32; *T*-value: 4.26 cluster size: 98 voxels; *P* < 0.001 uncorrected) after controlling for global atrophy. No clusters with decreased FC were found in patients compared to controls within this network. An identical analysis of the left dorsal homologue (left opIFG seed) likewise revealed no significant group differences.

Within the network seeded in the right MTG, sbvFTD patients showed decreased FC compared to HC individuals in a cluster localized in the right AG (MNI coordinates of the cluster peak: 46, −66, 34; *T*-value: 2.70 cluster size: 194 voxels; *P* = 0.003 uncorrected) after controlling for global atrophy. When the analysis was repeated for the left–ventral homologue (left MTG seed), sbvFTD patients exhibited reduced FC in a cluster centered on the left AG (MNI peak: −44, −60, 22; *T* = 5.48; cluster = 64 voxels; *P* = 0.002 uncorrected), and in a cluster in the medial frontal cortex (MNI peak: 0, 34, −20, cluster = 81, *T* = 4.54; *P* = 0.010 uncorrected) after controlling for global atrophy.

#### Brain-behaviour correlation in altered networks of sbvFTD

**Figure 2:**
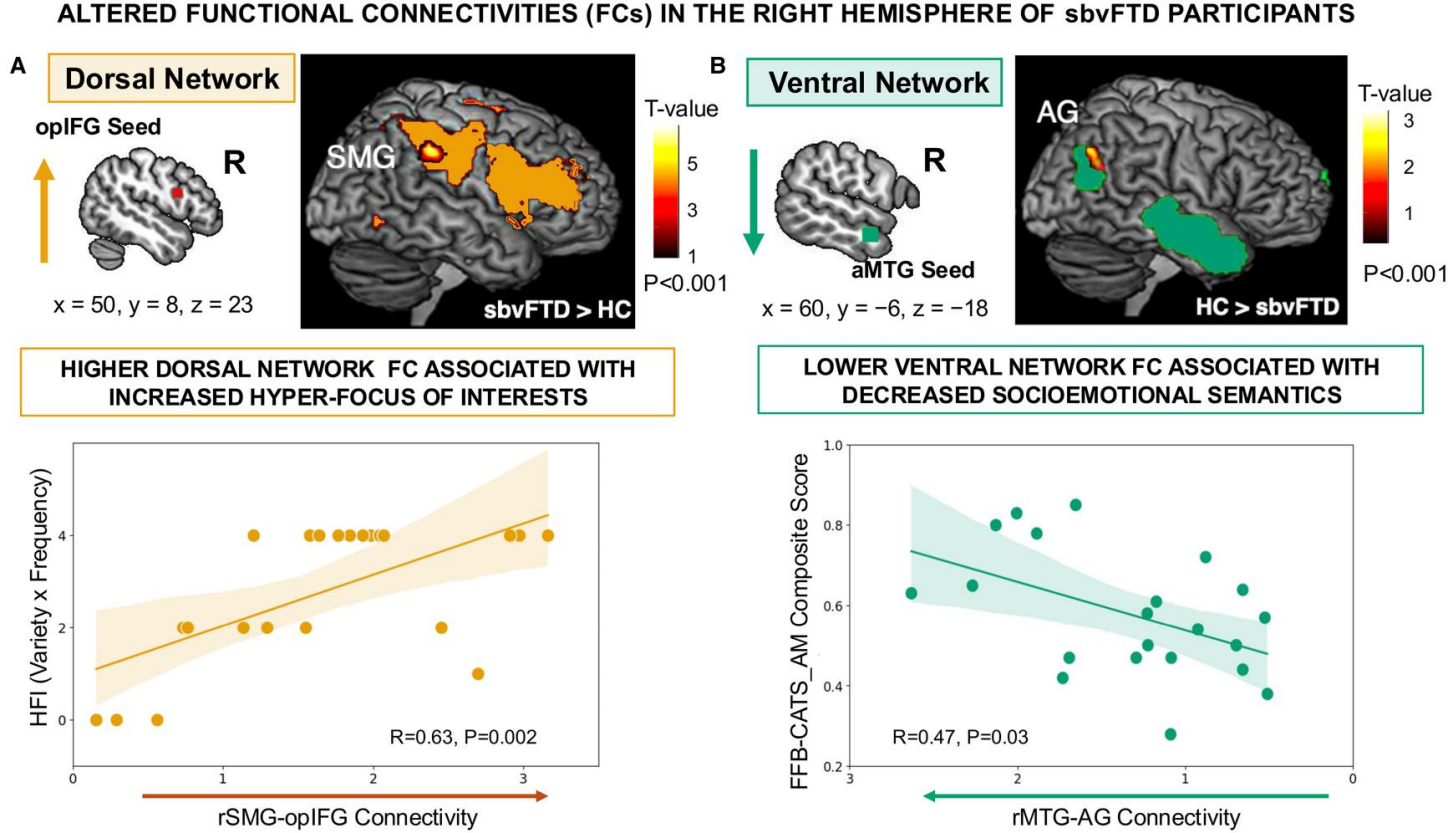
**Brain-behaviour correlations in the right hemisphere.** (**A**) Right dorsal network: higher scores of HFIs correlate with increased FC between the right SMG and the right opIFG (Pearson’s *r* = 0.63, *P* = 0.001) (**B**) Right ventral network: lower socioemotional semantic ability—indexed by the composite of FFB and CATS-AM—is associated with reduced FC between the right aMTG and right AG (Pearson’s *r* = 0.47, *P* = 0.03). Each dot represents one sbvFTD participant; the missing behavioural score were represented using averaged across the imputed data sets. Sample size: *N* = 22. sbvFTD, semantic behavioural variant frontotemporal dementia; FC, functional connectivity; HFI, Hyper-focus of Interests; SMG, supramarginal gyrus; opIFG, opercular inferior frontal gyrus; aMTG, anterior middle temporal gyrus; AG, angular gyrus; FFB, Famous-Faces Battery; CATS-AM, Comprehensive Affect Testing System-Affect Matching.

Correlation analyses on clusters with significant FC alterations showed a positive association between higher HFI scores and increased FC in the right dorsal network (right SMG within the network seeded in the right opIFG; *r* = 0.63, *P* = 0.002). Conversely, decreased FC in the right ventral network (right aMTG–right AG) was associated with poorer socioemotional-semantic performance (FFB and CATS-AM composite; *r* = 0.47; *P* = 0.03). The imputation analysis confirmed this positive association (beta = 0.12, SE = 0.053, *P* = 0.038). For visualization, the imputed values for the three participants with missing FFB data were averaged across datasets (Fig. 2). To validate hemispheric specificity, we correlated FC in the left ventral network (left aMTG–left AG) with verbal–semantic scores and found a similar positive association (BNT and PPVT composite: *r* = 0.61, *P* < 0.001; Supplementary Fig. 1). No brain-behaviour analysis was performed for the left dorsal network because we did not find any FC alterations compared to controls.

Post-hoc tests confirmed specificity: (i) higher HFI scores were not related to decreased FC in the right ventral network (*r* = 0.04, *P* = 0.85) or in its left-hemisphere counterpart (*r* = −0.26, *P* = 0.25); (ii) socioemotional semantics scores showed no correlation with increased FC in the right dorsal network (*r* = −0.32, *P* = 0.16), nor with decreased FC in the left ventral network (*r* = 0.40, *P* = 0.08); and (iii) verbal–semantic scores showed no correlation with increased FC in the right dorsal network (*r* = −0.07, *P* = 0.75) nor with decreased FC in the right ventral network (*r* = 0.30, *P* = 0.17).

## Discussion

This study provides novel neural insights into predominant right anterior–temporal lobe degeneration by showing that sbvFTD is marked by both increased and decreased FC, extending what can be inferred from atrophy alone. In patients with predominant right–ATL atrophy, higher connectivity within the right dorsal fronto-parietal network tracked a distinctive pattern of rigid, hyper–focused behaviours, whereas weaker connectivity in the right ventral temporo–parietal network predicted socioemotional semantic deficits. These double–dissociated results highlight complementary, but distinct, roles for the two networks in shaping symptom expression in sbvFTD. Consistent with contemporary models, we interpret these findings as graded hemispheric biases rather than absolute left–versus–right ‘modules.’ Because sbvFTD couples predominant right–ATL damage with systematic alterations in dorsal and ventral IPL connectivity, the syndrome offers a natural ‘lesion model’ for testing how the right IPL balances goal maintenance against re–orientation to salient new information. In this framework, patients’ HFIs can be viewed as an extreme form of goal persistence, providing a direct clinical window onto right–IPL network function. In the following paragraphs, we further examine the roles of these networks and cortical hubs in behaviour and cognition.

Our findings indicate that both the dorsal and the ventral networks exhibited significant FC alterations with subregions of the inferior parietal lobule (IPL), specifically the SMG (connected to the inferior frontal seed) and the AG (connected to the middle temporal seed). Recent findings suggest that both the left and right IPL modulate connectivity within their respective subregions in a domain-specific manner,^58^ with the left IPL consistently involved in language processing.^23,24,80-82^ Here, we focus on the right IPL and its possible role in the specific ‘rigid’ or ‘compulsive’ behaviours described in sbvFTD, now described under the rubric of HFI behaviour.^28^ Functional imaging, electrophysiological, and lesion-based studies have implicated the right IPL as integral to a system that facilitates flexible behavioural reconfiguration between two distinct operational modes.^55,58,83^ Damage to the right IPL results in deficits related to (i) maintaining attentive control over current task goals and (ii) responding to salient new information or alerting stimuli in the environment. These impairments contribute to hemineglect, a classical syndrome often observed following lesions in this region.^55^ Additionally, evidence from neurodevelopmental studies indicates that the right IPL selectively responds to both motoric and perceptual representations of actions, as well as socioemotional components of visual stimuli during action observation.^53,54^ Previous parcellation studies in humans have delineated the right IPL and revealed distinct organizational patterns compared to nonhuman primate brains. These findings underscore the multidimensional role of the right IPL in cognition and behaviour.^84-87^ The forthcoming sections will elaborate on the specific engagement of each segment of the IPL within each intrinsic network.

### Right dorsal fronto-parietal network and its role in rigid, restricted behaviours

The lack of understanding of the neural mechanisms underlying this common symptom of hyper-focus on restricted, rigid interests in sbvFTD is a critical gap in the field.^6,19^ From a behavioural perspective, previous studies propose that semantic loss and shifts in the hedonic system might underlie these factors,^14,37,38,40^ but these theories were not empirically tested nor anatomically substantiated. One study found that individuals with SD used more efficient visual search strategies than HCs when the number of distractors increased.^83^ The study revealed that conjunction search involving the highest number of distractors correlated with grey matter volume in the dorsal fronto-parietal network, which was spared in the SD group. The researchers hypothesized that the increasing attention skills observed in SD might arise from alterations in network connectivity due to decreased ventral stream information flow and enhanced function, but not altered structure, of grey matter in the dorsal fronto-parietal network. However, their structural neuroimaging analyses did not allow them to directly show the FC alterations nor test hemispheric contributions.^83^

In the present study, we show increased FC in the right dorsal fronto-parietal network in sbvFTD and show that it correlates with symptoms attributable to HFIs. Previous work investigating the role of the IFG-SMG connectivity supports this hypothesis by showing its involvement in sustaining attention, detecting salient or novel events, phasic alerting, and task-set switching,^55,88-91^ and highlighting its contributions to behaviour.^53,54,56-58,92-94^ Further functional segregation analyses identified five different IFG clusters related to their connections to other networks, specifically the opIFG and SMG, which are linked to task-specific attentional load and task execution.^57^ Moreover, increased FC in this network has been attributed to heightened task-related activity and increased attentional load when targets and distractions are equal,^95^ rule acquisition,^96^ and activity modulation based on the outcome valence of choice tasks.^97^

Regarding the ‘impact of valence’, stronger activity increases were observed in a reward-seeking condition compared to a punishment-avoidance condition, indicating a stronger responsiveness to ‘better than expected’ outcomes.^97^ Finally, Uddin *et al*. (2021)^92^ underscored the role of this network in behavioural flexibility, while highlighting the contributions of other anatomical areas, such as the orbitofrontal cortex and dorsomedial striatum, especially in reward-related behavioural flexibility. Overall, these regions are crucial for maintaining focus on a task in the face of distraction and, conversely, for flexibly switching to new external demands when necessary for optimal behaviour guidance.^55^ In the case of sbvFTD, this system might lean towards ‘maintaining the task’ rather than ‘switching to new stimuli’, which patients may have difficulty interpreting, especially if the system is overactive, as indicated in this study.

In this framework, structural damage and cognitive dysfunction in the right ventral semantic network in sbvFTD would shift interest away from activities involving nuanced, socially relevant knowledge, while relative structural sparing and increased connectivity in the dorsal circuit would increase attention to activities that require less interpersonal socioemotional engagement, such as collecting, writing, or exercising.^6,33,36^

### Right ventral temporo-parietal network and its role in socioemotional semantics

We observed a correlation between lower FC in the aMTG-AG connection within the right ventral network and poorer performance on cognitive tests targeting specific semantic processes, such as famous face identification and emotion expression recognition. This finding indicates a role for this network in socioemotional semantics, relative to intact perception skills. A large body of functional neuroimaging literature depicts a major role for the AG in processing concepts rather than percepts when interfacing perception-to-recognition-to-action.^25,98-100^ More specifically, the AG emerges as a cross-modal hub where converging multisensory information is combined and integrated to comprehend and give meaning to events, manipulate mental representations, solve familiar problems, and reorient attention to relevant information.^101^ The pathway linking the aMTG and AG, connected by the middle longitudinal fasciculus,^102,103^ serves a distinct function in semantic processing.^13,24,25,101,104^ This association has been studied on the left side,^24^ supporting functions related to the control and manipulation of verbal and object-based semantics within the context of the large-scale default network. Specifically, individuals with svPPA showed decreased FC in the left ventral semantic network, correlated with weak word knowledge. While the right-sided network in sbvFTD has not been extensively studies, evidence consistently points to its hub regions being involved in socioemotional semantics as well.^13,15,105^ Our findings contribute to the literature by confirming existing knowledge on ventral semantic networks and providing anatomical evidence for the clinical symptoms commonly seen in sbvFTD. This supports the use of more precise terminology that reflects the affected neural mechanisms.

It is noteworthy that the combination of damaged and spared neural networks, along with both decreased and increased connectivity, is necessary to produce such unique clinical manifestations. These manifestations likely occur in the early stage of the disease and eventually recede as it becomes more widespread. Such phenomena have also been described in the context of new talents emerging or changes in artistic expression in focal neurodegenerative diseases.^83,105-107^ Neuroimaging studies showing similar network alterations in svPPA suggest that a neurodegenerative lesion to the anterior temporal lobes leads to a functional reorganization of the brain networks.^108^ Future studies employing novel methodologies are warranted to elucidate how neural dynamics alter in response to focal neurodegeneration over the course of the disease, leading to changes in clinical manifestations over time.

### Limitations

The study has several limitations. First, our sample size was relatively small (*n* = 22), which may limit the generalizability of the results. However, given the rarity of this disease, the cohort is large compared to other published reports. We also created a highly specific patient cohort by excluding individuals with severe global cognitive decline and greater disease severity, focusing on those with predominant atrophy in the temporal lobe, as measured by an objective atrophy index. All patients were recruited from a prospective, multidisciplinary, longitudinal observational study. Some data were missing for certain tests, often because test batteries were updated over time or participants became fatigued. We addressed these gaps by imputing missing values using well-established relationships, particularly between performance on famous people knowledge tests and volume loss of the right temporal lobe, and by estimating scores from detailed examiner notes. Because no validated objective tests exist for measuring engagement in hyper-focused behaviours, we relied on chart reviews. To mitigate potential biases, four independent raters reviewed the case notes and assessed the frequency and variety of HFI behaviour. Although not standardized, this approach was a necessary adaptation that allowed inclusion of this important behavioural measure. Finally, this study focused on the right hemisphere counterparts of the two well-established language networks, the dorsal fronto-parietal and ventral temporo-parietal networks.^24,32^ However, other work has shown a more complex pattern in svPPA, with both decreases and increases in connectivity across additional networks—for example, reduced aMTG–DMN and aMTG–Broca connectivity but heightened aMTG–dorsal–attention, dorsal–attention–visual–association, and aMTG–planum temporale connectivity.^108,109^ Although we focused on these two networks based on prior studies, we cannot exclude the possibility that alterations in other circuits may contribute differently to behaviours commonly observed in sbvFTD. Our findings clarify the heightened attention and task-execution component of HFI behaviour, but future studies are needed to test whether these behaviours are linked to additional networks and to identify circuits underlying the narrowness of interests and altered reward or motivation. These questions are beyond the scope of the present study but remain important directions for future research.

## Conclusion

This study provides novel neural insights into the poorly understood symptoms associated with predominant rATL degeneration. Our findings uncover alterations of both increased and decreased network connectivity in sbvFTD, extending beyond traditional atrophy-based approaches. These results deepen our understanding of the neurobiological basis of clinical manifestations in focal neurodegeneration.

## Supplementary Material

fcaf251_Supplementary_Data

## Data Availability

Upon journal publication, individual-level data from this study will be shared in an access-controlled FAIR Alzheimer’s Disease Data Initiative AD Workbench repository at https://fair.addi.ad-datainitiative.org. Descriptive statistics, *t*–tests, χ²–tests, and Pearson correlations were executed in Python 3.10.9 with the standard scientific stack (pandas 2.2.2, numpy 1.26.4, scipy 1.12.0, and statsmodels 0.14.0). Structural MRI processing was performed with CAT12 v12.8 running in SPM12 (r7771) under MATLAB 2021b. Functional MRI preprocessing was automated via a Python 3.10.9 wrapper that includes FSL v6.0.7, SPM12, ANTs v2.4.4, and AFNI v23.3.09, while ComBat harmonization was carried out in R using the open–source package.
