# Supplementary material for: Network changes associated with right anterior temporal lobe atrophy: insight into unique symptoms
Source: Brain Commun. 2025 Jun 24;7(4):fcaf251. doi: 10.1093/braincomms/fcaf251 (PMC12238713; doi:10.1093/braincomms/fcaf251)
Supplement: fcaf251_Supplementary_Data [file fcaf251_supplementary_data.docx]

**Supplementary Table 1 Cognitive and behavioral data**

| Values reported as mean (SD) | **N** | **sbvFTD** | **N** | **HC** | **P value** |
| --- | --- | --- | --- | --- | --- |
| ***Attention*** | | | | | |
| Digit Span Forward | 20 | 7.1 (1.2) | 28 | 7.5 (1.1) | n.s. |
| ***Executive Functioning*** |  |  |  |  |  |
| Digit Span Backward | 19 | 5.1 (1.0) | 38 | 5.8 (1.4) | n.s. |
| Stroop (correct in 60 s) | 18 | 79.3 (20.1) | 40 | 95.0 (5.6) | n.s. |
| Design Fluency | 18 | 8.6 (3.8) | 39 | 13.2 (3.6) | 0.02 |
| Trials Time | 18 | 39.5 (16.8) | 40 | 26.7 (15.3) | 0.03 |
| ***Calculation*** | | | | | |
| Calculation | 19 | 4.5 (0.6) | 31 | 4.7 (0.5) | n.s. |
| ***Rule Violation (RV)*** | | | | | |
| Verbal Fluency – Phonemic RV | 21 | 0.2 (0.7) | 40 | 0.4 (0.5) | n.s. |
| Verbal Fluency- Categoric RV | 21 | 0.06 (0.2) | 40 | 0 | n.s. |
| Design Fluency RV | 18 | 0.3 (0.6) | 40 | 0.1 (0.3) | n.s. |
| ***Visuospatial Processing*** | | | | | |
| Modified Rey–Osterrieth Figure - copy | 20 | 15.6 (0.7) | 41 | 15.3 (1.0) | n.s. |
| VOSP Number Location | 20 | 9.4 (1.9) | 39 | 9.6 (0.7) | n.s. |
| ***Episodic Memory*** | | | | | |
| CVLT 30’ short delay free recall | 20 | 4.9 (2.7) | 6 | 8.8 (0.4) | <0.001 |
| CVLT 10’ long delay free recall | 20 | 3.5 (2.9) | 6 | 8.4 (0.5) | <0.001 |
| CVLT Recognition | 20 | 7.3 (2.4) | 6 | 8.6 (0.5) | <0.001 |
| Modified Rey–Osterrieth Recognition | 20 | 0.8 (0.4) | 39 | 0.7 (0.4) | n.s. |
| Modified Rey–Osterrieth- 10 min delay | 20 | 6.7 (4.5) | 39 | 11.3 (2.5) | 0.02 |
| ***Language*** |  |  |  |  |  |
| Repetition | 19 | 2.9 (0.2) | 39 | 3.0 (0.0) | n.s. |
| WRAT Reading | 18 | 59.1 (5.6) | 23 | 65.8 (1.6) | 0.005 |
| Syntax Comprehension | 19 | 4.5 (0.7) | 16 | 4.9 (0.2) | 0.02 |
| Verbal Agility | 19 | 5.4 (0.9) | 17 | 5.8 (0.3) | n.s. |
| Verbal Fluency – Phonemic | 21 | 11.4 (3.7) | 40 | 18.1 (2.4) | <0.001 |
| Verbal Fluency - Categoric | 21 | 13.9 (4.2) | 40 | 23.8 (1.9) | <0.001 |
| Boston Naming Test | 19 | 8.4 (3.6) | 40 | 14.5 (0.7) | <0.001 |
| Peabody Picture Vocabulary Test | 21 | 10.2 (3.1) | 16 | 15.7 (0.3) | <0.001 |
| Pyramids and Palm Trees Pictures (%) | 20 | 0.9 (0.1) | 30 | 0.9 (0.01) | <0.001 |
| ***Face Perception & Person Specific Knowledge*** | | | | | |
| CATS Face Matching | 20 | 11.5 (0.8) | 15 | 11.8 (0.3) | n.s. |
| Famous Faces Naming % | 18 | 0.3 (0.2) | 26^$^ | 0.7 (0.2) | <0.001 |
| Famous Faces Familiarity % | 16 | 0.7 (0.2) | 26^$^ | 0.8 (0.1) | <0.001 |
| Famous Faces Semantic Association % | 17 | 0.7 (0.2) | 26^$^ | 14.7 (0.9) | <0.001 |
| ***Social Function & Emotion*** | | | | | |
| CATS Affect Matching | 20 | 10.4 (3.0) | 15 | 14.6 (1.5) | <0.001 |
| Dynamic Affect Recognition Test | 8 | 6.7 (1.9) | 71^#^ | 10.1^#^ (2.1) | <0.001 |
| TASIT- Emotion Evaluation Test | 7 | 6.0 (2.3) | 6 | 10.5 (1.3) | <0.001 |
| TASIT- SI-M Sarcastic | 15 | 5.9 (4.7) | 6 | 19.3 (1.2) | <0.001 |
| TASIT- SI-M Sincere | 15 | 16.6 (2.5) | 6 | 17.3 (2.6) | n.s. |
| Cognitive Theory of Mind | 16 | 14.5 (1.6) | 5 | 15.5 (0.5) | n.s. |
| Emotional Theory of Mind | 10 | 12.3 (1.3) | 5 | 15.7 (0.3) | <0.001 |
| IRI- Empathic Concern | 14 | 20.3 (8.6) | 18 | 27.8 (5.2) | <0.001 |
| IRI- Perspective Taking | 14 | 14.3 (6.0) | 18 | 24.1 (7.0) | <0.001 |
| RSMS- Expressive Behavior | 16 | 5.1 (3.0) | 5 | 22.6 (5.1) | <0.001 |
| RSMS- Self Presentation | 16 | 10.3 (5.1) | 5 | 26.4 (5.4) | <0.001 |

*= Categorical variables; ^#,$^ = Since the present control group was not tested on these variables, we used data from Younes et al. (2022) (^$^) and an independent group of 71 older healthy participants (^#^) to test differences in these scores between a control population and the sbvFTD patients enrolled in this study. CATS: Comprehensive Affect Testing System; IRI = Interpersonal Reactivity Index; TASIT = The Awareness of Social Inference Test; RSMS = Revised Self-Monitoring Scale; SI-M = Social Inference- Minimal.

N represents the number of participants for which tests were available.

“Trials Time” represents the time that participants take to finish the modified trials test.

**
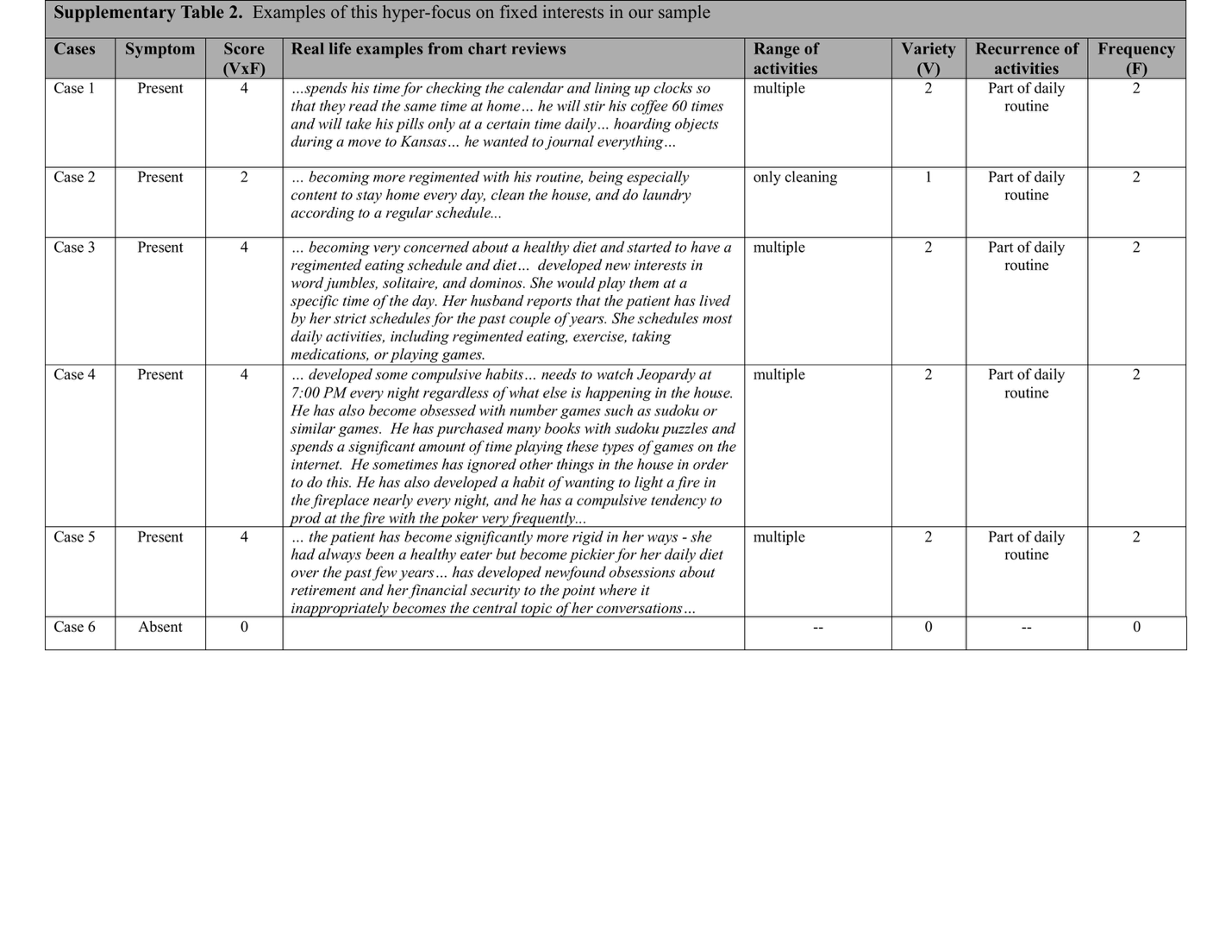
**

**
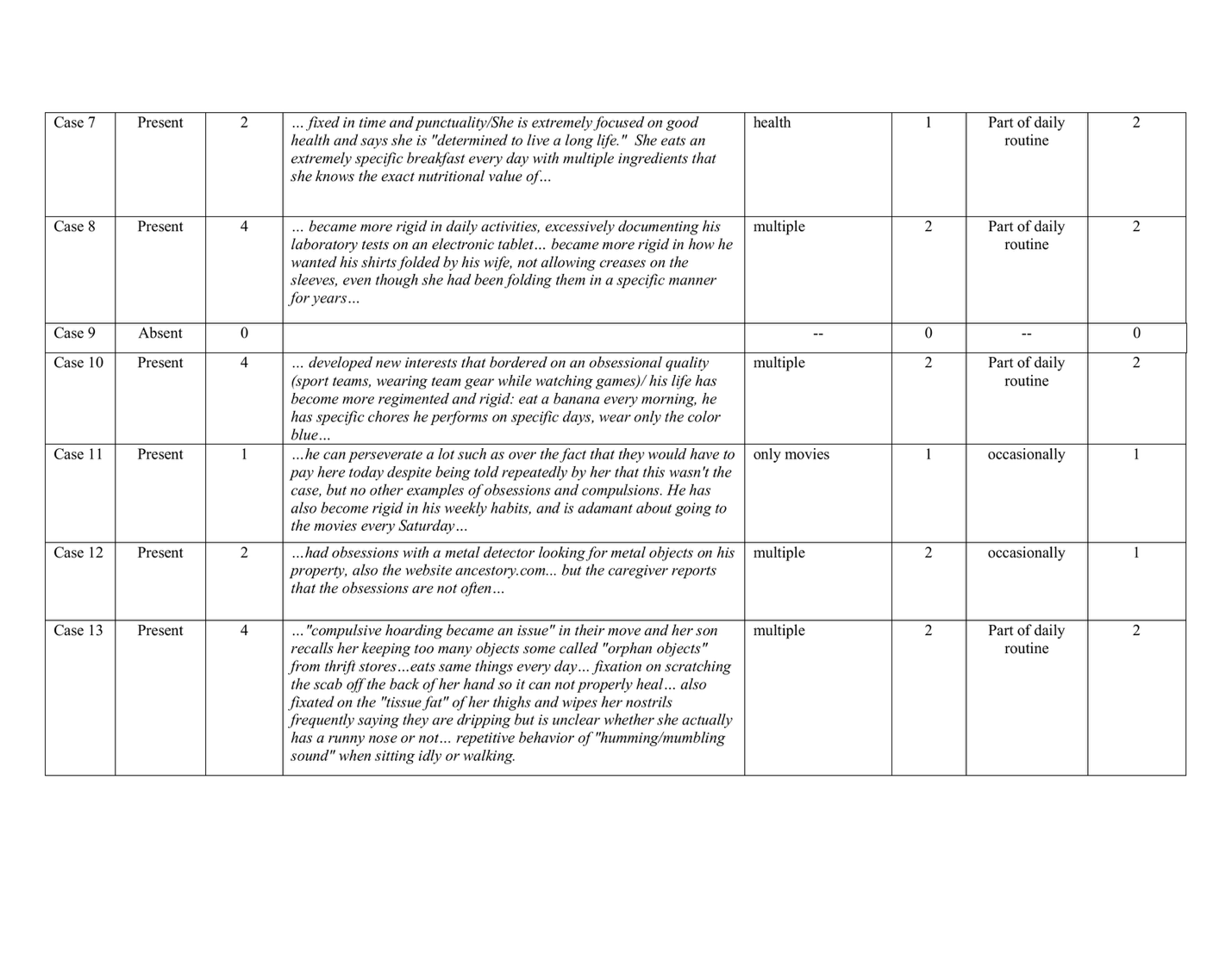
**

**
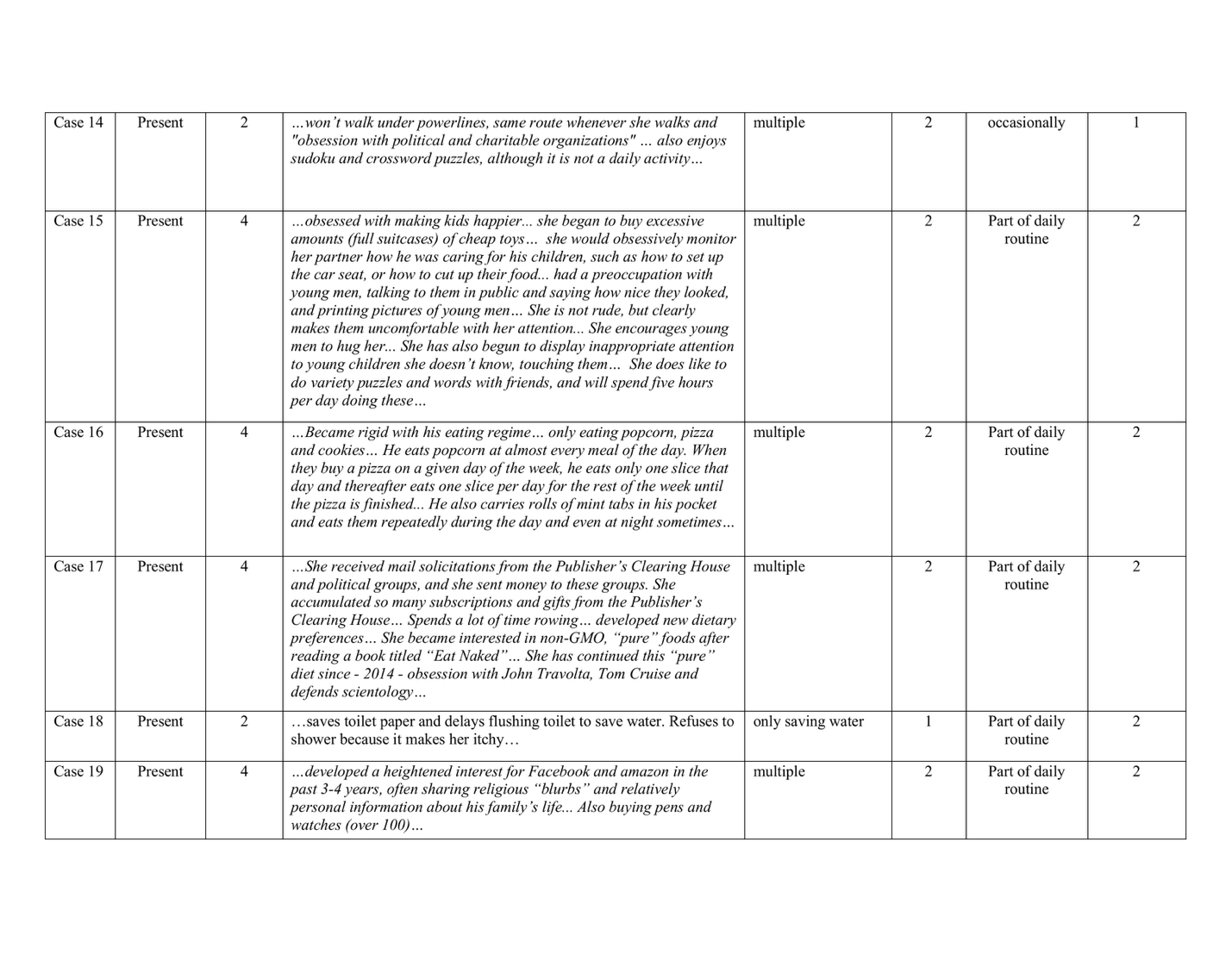
**

**
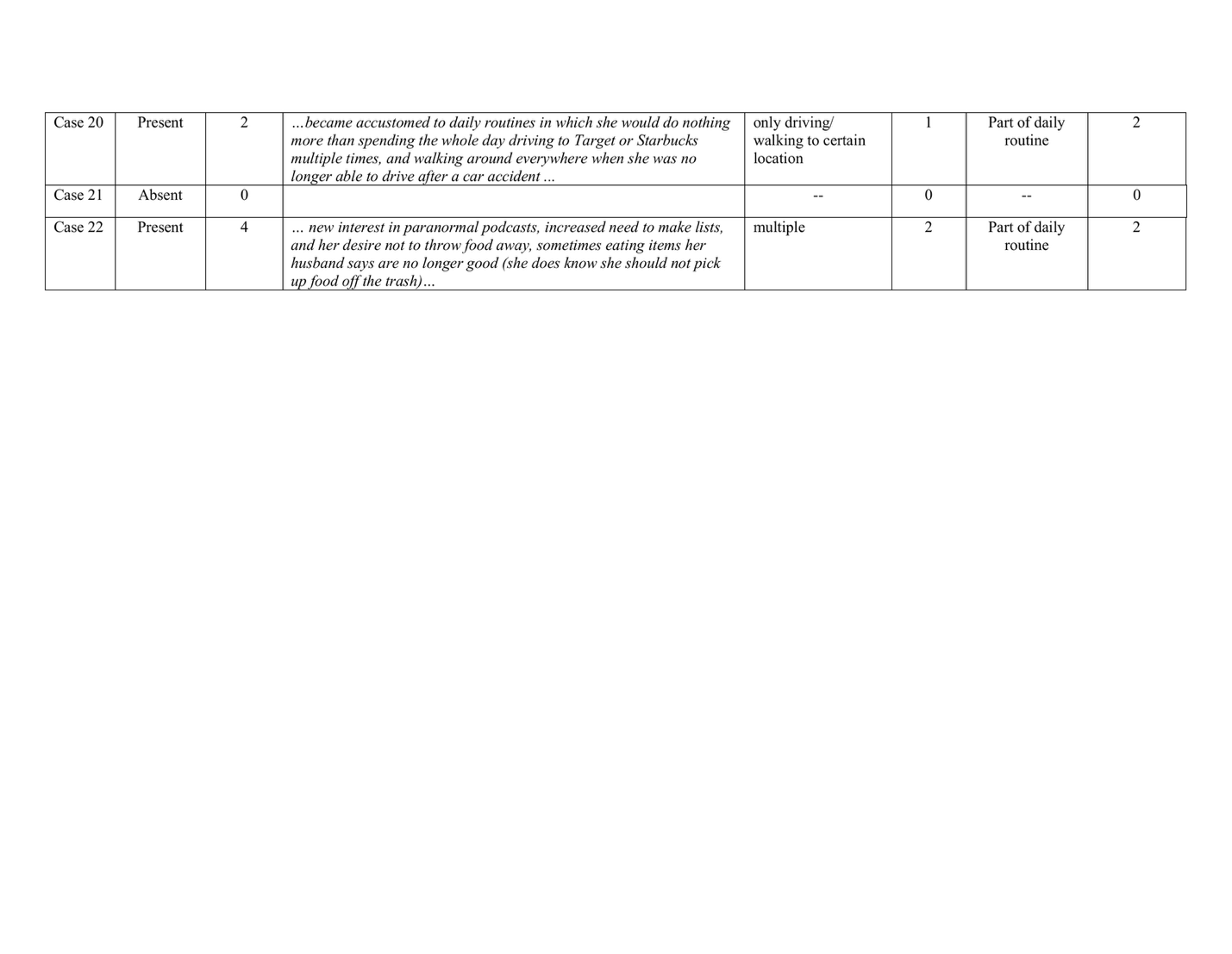
**

**Methods. Missing data handling**

Three patients had documented clinical descriptions of impairment severity on the famous faces battery (FFB) but lacked exact scores for the three tasks. To estimate these missing scores, we applied Multiple Imputation by Chained Equations (MICE)^1^ using the *mice* package in R. Prior evidence links bilateral temporal lobe involvement in face processing, with right temporal regions associated with recognition and left temporal regions with naming^2–4^. Our data support this association, showing positive correlations between the FFB naming component and atrophy in both the left (R = 0.56, *P* = 0.007) and right (R = 0.66, *P*= 0.001) temporal lobes. However, the triplet (R = 0.42, *P*= 0.046) and recognition (R = 0.50, *P*= 0.025) components correlated only with the right temporal atrophy. Consequently, the total FFB score correlated only with right temporal atrophy (R = 0.65, *P*= 0.002), which was used as the predictor for imputing the FFB total score. Predictive Mean Matching ensured that each imputed value closely matched the distribution of observed scores. For each imputation iteration, the composite score was recalculated, and the distribution of imputed data was inspected to confirm alignment with observed data.

Twenty imputed datasets were generated, with simple regression models estimated and pooled according to Rubin’s rules^5^. The resulting coefficients and standard errors were combined with **mice::pool**, so all 20 imputations contributed to the final effect size and P‑value. For Figure 2, the mean of each participant’s 20 imputed FFB totals is plotted solely for visualization.

**REFERENCES**

1. Buuren, S. van & Groothuis-Oudshoorn, K. mice: Multivariate Imputation by Chained Equations in R. *J. Stat. Softw.* **45**, 1–67 (2011).

2. Ding, J. *et al.* A unified neurocognitive model of semantics language social behaviour and face recognition in semantic dementia. *Nat. Commun.* **11**, (2020).

3. Snowden, J. S., Thompson, J. C. & Neary, D. Knowledge of famous faces and names in semantic dementia. *Brain J. Neurol.* **127**, 860–872 (2004).

4. Ralph, M. A. L., McClelland, J. L., Patterson, K., Galton, C. J. & Hodges, J. R. No Right to Speak? The Relationship between Object Naming and Semantic Impairment:Neuropsychological Evidence and a Computational Model. *J. Cogn. Neurosci.* **13**, 341–356 (2001).

5. Rubin, D. B. *Multiple Imputation for Nonresponse in Surveys*. (Wiley, 1987). doi:10.1002/9780470316696.

**Supplementary Table 3.** Coordinate of the local maxima within the gray matter profile of atrophy

|  | **MNI Coordinate** | | | **Cluster Extent (mm^3^)** | **P (FWE)** | **Max T** |
| --- | --- | --- | --- | --- | --- | --- |
|  | **X** | **Y** | **Z** |  |  |  |
| **Right brain regions** |  |  |  | 108072 |  |  |
| Temporal pole | 22 | 3 | -44 |  | 0.000 | 22.22 |
| Amygdala | 26 | 4 | -21 |  | 0.000 | 20.31 |
| Entorhinal cortex | 26 | -3 | -37 |  | 0.000 | 19.45 |
| Inferior temporal gyrus | 46 | 4 | -43 |  | 0.000 | 17.99 |
| Anterior Insula | 43 | 10 | -4 |  | 0.000 | 17.47 |
| Hippocampus | 34 | -18 | -9 |  | 0.000 | 17.27 |
| Fusiform gyrus | 39 | -20 | -27 |  | 0.000 | 16.53 |
| Middle temporal gyrus | 56 | 2 | -22 |  | 0.000 | 16.21 |
| Superior temporal gyrus | 44 | -6 | -14 |  | 0.000 | 15.84 |

P values (P) and maximum T statistics (Max T) in the local maxima within the main significant cluster.

P values were controlled for family wise error - FWE.

**Supplementary Figure 1**


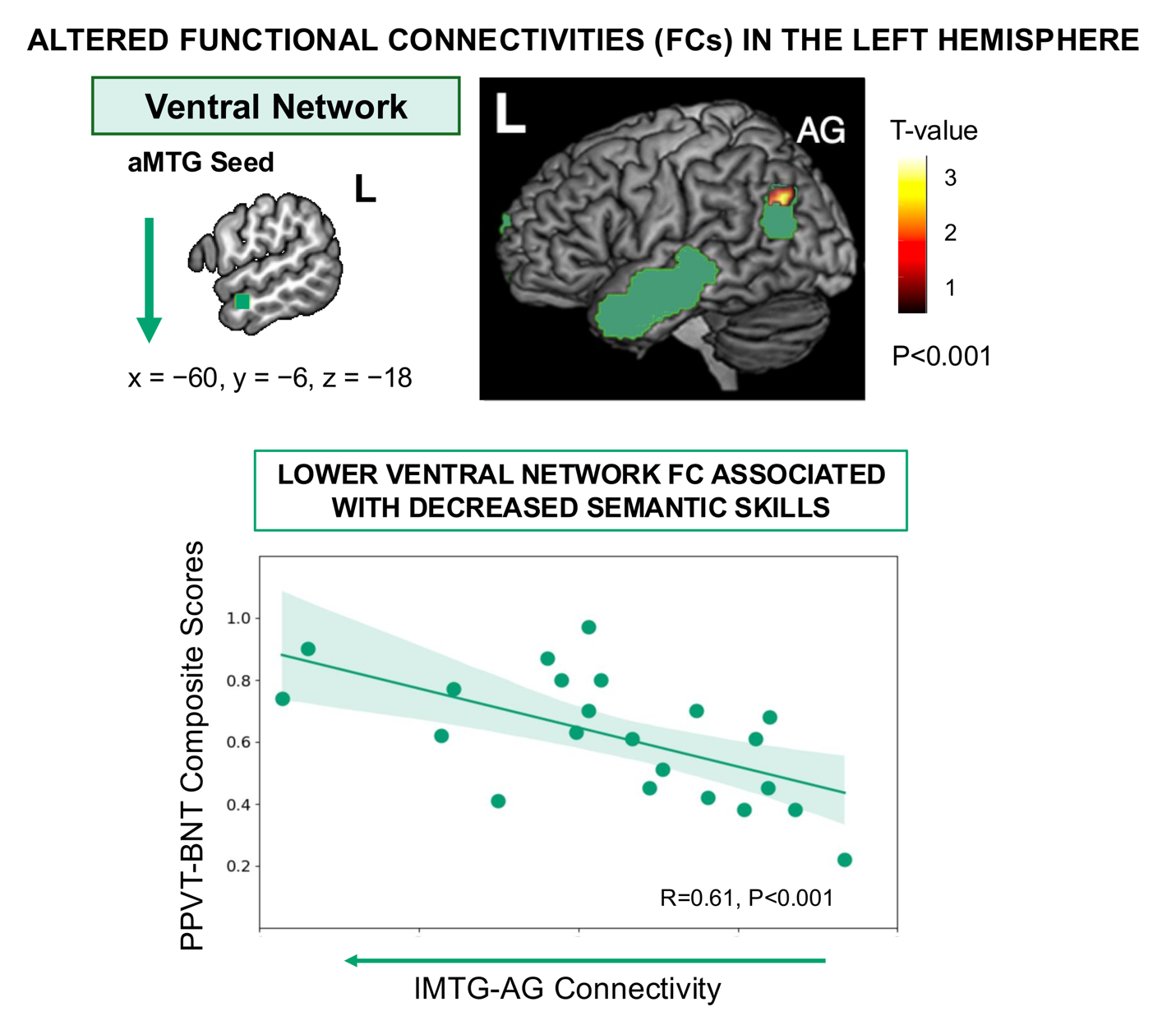


**Supplementary Figure 1: Brain–behavior correlations in the left ventral network.** Reduced scores of the composite verbal-semantic score (Boston Naming Test + Peabody Picture Vocabulary Test) in sbvFTD participants correlate with lower functional connectivity (FC) between the left anterior middle temporal gyrus (aMTG) and left angular gyrus (AG) (Pearson’s *r* = 0.61, *P* < 0.001). Each dot represents one sbvFTD participant. Sample size: N = 22.

Abbreviations: sbvFTD, semantic-behavioural variant frontotemporal dementia; FC, functional connectivity; aMTG, anterior middle temporal gyrus; AG, angular gyrus; L, left; BNT, Boston Naming Test; PPVT, Peabody Picture Vocabulary Test.
